# Development of buspirone hydrochloride-loaded long-acting microneedles for management of anxiety disorders

**DOI:** 10.1007/s13346-025-01803-1

**Published:** 2025-02-14

**Authors:** Tanvi Karve, Nisha Shrestha, Thomas Kipping, Ajay K. Banga

**Affiliations:** 1 Center for Drug Delivery Research, Department of Pharmaceutical Sciences, College of Pharmacy, Mercer University, 3001 Mercer University Drive, Atlanta, GA 30341 USA; 2 MilliporeSigma a Business of Merck KGaA, Frankfurter Strasse 250, 64293 Darmstadt, Germany

**Keywords:** Microneedles, Transdermal, Buspirone, Long-acting, Sustained drug delivery, Anxiety

## Abstract

Buspirone hydrochloride (BSP) is an anxiolytic agent approved for the management of anxiety disorders. The current US-FDA approved medications of BSP are administered via the oral route, which is linked to several disadvantages such as low oral bioavailability and low half-life necessitating multiple daily doses. For chronic diseases such as anxiety disorders, where long-term or lifelong management is often required, these factors impact patient compliance and treatment adherence. The present study offers an alternative treatment approach by investigating the feasibility of sustained transdermal delivery of BSP via long-acting microneedles (MNs). Needle-tip-loaded MNs were fabricated via micro-molding technique using various grades of poly-vinyl alcohol (PVA) namely 4–88, 8–88, 18–88, and 26–88. These MNs were compared using characterization techniques such as Parafilm^®^ insertion testing, scanning electron microscopy (SEM), confocal microscopy, Fourier transform infrared spectroscopy (FTIR), and histological evaluation of MNs-treated human skin. The effect of different grades of PVA on the structural and mechanical properties of the fabricated MNs was evaluated. Further, in vitro release and permeation tests were conducted to assess the drug release patterns and transdermal delivery across dermatomed human skin over 7-day study periods. The highest release (5507.37 ± 456.88 µg/cm^2^) and delivery (4705.42 ± 634.57 µg/cm^2^) were observed from PVA 4–88, with significant differences among the PVA grades based on their properties. Notably, all four types of the fabricated PVA MNs crossed the daily and weekly therapeutic targets for the systemic delivery of BSP. Overall, this study established the feasibility of sustained delivery of BSP across the skin using PVA MNs for the management of anxiety disorders.

**Graphical Abstract:**

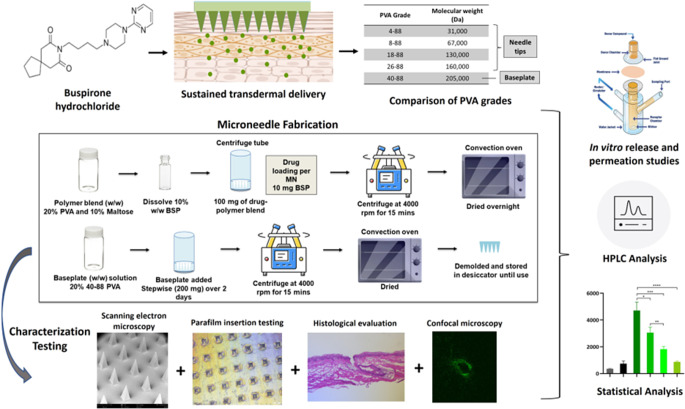

## Introduction

Anxiety disorder is the most common psychological illness in the United States, affecting nearly 20% of the total population and nearly 300 million people worldwide [1, 2]. Anxiety disorders are characterized by motor tension, palpitations, autonomic hyperactivity, apprehensive expectation, avoidance behavior, and vigilance and scanning tendencies [3, 4]. Moreover, anxiety disorders have a high co-occurrence rate with other psychological disorders such as depression, bipolar disorder, irritable bowel syndrome, adult attention-deficit/hyperactive disorder (ADHD), and substance abuse [1, 5]. Estimated to have the highest lifestyle prevalence, anxiety disorders significantly impact quality of life. Clinical research indicates that individuals with anxiety suffer from functional impairment with social, physical, and occupational implications [6]. Hence, there is a high demand for long-acting, patient compliant drug products for the management of anxiety disorders. At present, the management of anxiety disorders is supported by various approved medication classes such as selective serotonin reuptake inhibitors (SSRIs), benzodiazepines, norepinephrine reuptake inhibitors, and anxiolytic agents [7].

BSP (MW 422 g/mol, logP 1.66) is a hydrophilic, small molecule therapeutic that belongs to the class of anxiolytics [8]. Although the exact mechanism of BSP is unknown, its pharmacology is mediated via serotonin 5-HT receptors [9]. As opposed to most other anti-anxiety medication classes, BSP is free from side effects such as sedation/drowsiness, insomnia, and impaired cognitive and motor functions [10]. Furthermore, it has also been reported to treat some side effects caused by other classes such as SSRIs [11]. BSP is FDA-approved for the management of anxiety via the oral route of administration in the form of tablets with varying strengths upto 30 mg [12, 13]. However, this treatment option suffers from various route-specific disadvantages. The oral bioavailability of buspirone hydrochloride is extremely low (4%) because the drug undergoes extensive first-pass metabolism. Moreover, the half-life of the drug is only 2–4 h, necessitating multiple daily doses [14]. These factors negatively impact patient compliance and can restrict adherence to the dosing regimen. Hence, the exploration of alternative routes of administration to address these drawbacks is of significance.

The present study offers transdermal delivery as an attractive alternative. The skin, being the largest organ with an average surface area of 2 m^2^, makes it an accessible and non-invasive route of administration [15]. Due to the skin’s tightly packed lipidic structure, a majority of therapeutically active compounds are unable to passively permeate into the skin’s layers [16]. To enhance the delivery of such actives, various chemical penetration enhancers and physical enhancement techniques have been explored [17, 18]. MNs is the most extensively researched approach among the existing physical enhancement techniques. MN transdermal delivery systems (MN-TDS) are an array of micron-sized needles. Upon insertion into the skin, these needles puncture the stratum corneum and epidermal layers, facilitating the passage of drugs from the MN-TDS to the skin’s microvasculature and further into the systemic circulation [19]. They are minimally invasive and pain-free systems, offering high bioavailability and ease of self-administration. Depending on patient needs, MN-TDS can be modulated for various release kinetics and mechanisms, such as rapid release (< 1 min) and programmable burst release (based on feedback loops). For chronic conditions requiring treatment over long periods, long-acting MN-TDS have been under active exploration in the past decade due to their advantages over conventional oral and parenteral therapies [20].

Polymeric, dissolving MNs are one of the most versatile types of MNs, with research interest in them increasing significantly over the past two decades [21]. PVA is a synthetic, biodegradable, slow-dissolving polymer extensively researched for fabricating TDS, including dissolving MNs [22, 23]. It is an inert and non-toxic polymer which is generally recognized as safe (GRAS) by the US FDA for a variety of pharmaceutical applications. Due to its high molecular weight, the PVA polymer itself does not significantly permeate across the skin’s layers and gets removed naturally as a part of skin turnover [24–26]. PVA’s water-soluble nature allows the controlled degradation of the polymer matrix when in contact with biological fluids, aiding in the design of delivery systems [27]. Moreover, its dissolution characteristics eliminate the need for removing the TDS and its nontoxicity and biocompatibility provide low immunogenicity [28, 29]. Additionally, the low material cost of PVA, combined with the high reproducibility of the micro-molding technique, makes this approach economically viable [30]. Although PVA by itself provides excellent mechanical strength, it is also explored in conjugation with other materials, such as carboxymethyl cellulose, polyvinylpyrrolidone, and chitosan, to provide flexibility to the structure [31–33].

The present study aims to fabricate and evaluate PVA-based, polymeric, slow-dissolving, needle-tip-loaded MNs for the sustained transdermal delivery of BSP. Four different grades of PVA (4–88, 8–88, 18–88, and 26–88) were utilized to fabricate the needle-tips of the MN-TDS. These grades possess the same degree of hydrolysis of the acetate ester functionality (represented by 88%) but differ in the molecular weights. Maltose, a disaccharide, was used in conjugation with PVA to improve the mechanical strength of the MN-TDS. The fabricated PVA MN-TDS were compared via characterization studies to understand the influence of the grades on the mechanical strength, structural integrity, and insertion capability of the MNs. Further, the in vitro performance of the fabricated MNs was tested. Drug release and permeation studies were conducted over 7-day study periods to compare the performance of the different grades of PVA. Overall, the present study establishes the feasibility of sustained, long-acting transdermal delivery of BSP using polymeric slow-dissolving PVA MN-TDS for the management of anxiety disorders.

## Materials and methods

### Materials

Buspirone hydrochloride was purchased from Sigma-Aldrich (Burlington, MA). Stainless-steel master structures (pyramidal, 10 × 10 array, base 150 μm x 150 μm, height 500 μm) were purchased from Micropoint Technologies Pte Ltd (Singapore). Silicone elastomer Sylgard^®^ base and curing agent was obtained from Dow Corning (MI, USA). Different PVA types/grades were obtained as gift samples from Merck KGaA (Darmstadt, Germany) including PVA 4–88 (M_W_ = 31 kDa), PVA 8–88 (M_W_ = 67 kDa), PVA 18–88 (M_W_ = 130 kDa), PVA 26–88 (M_W_ = 160 kDa), and PVA 40–88 (M_W_ = 205 kDa). Dermatomed human cadaver skin was obtained from a tissue bank. Fluoresoft-0.35%^®^ was obtained from Holles Laboratories Inc. (Cohasset, MA, USA). Phosphate buffered saline (PBS, pH 7.4) and propylene glycol were obtained from Fisher Scientific (Fisher BioReagent, NJ, USA) and Ekichem (Joliet, IL, USA), respectively. HPLC-grade solvents were obtained from Pharmaco-aper (Brookfield, CT, USA).

### Methods

#### Quantitative analysis

An RP-HPLC method was developed for the quantitative analysis of BSP. An Eclipse Plus C18 column (3.5 μm, 2.1 × 150 mm) at 25 °C was used with a Waters Alliance 2695 separations module (Milford, MA, U.S.A.). The mobile phase was optimized to a 30:70 ratio of acetonitrile with 0.1% trifluoroacetic acid and 10 mM of disodium phosphate buffer (pH 5.5) at a flow rate of 1 mL/min. The injection volume was fixed at 10 µL. The retention time was observed as 3.5 min at 240 nm. The calibration curve of BSP was prepared in PBS using serial dilutions in the range of 0.1 to 50 µg/mL. Further, the method was validated for linearity, intraday-interday accuracy-precision, limit of quantitation (LOQ), and the limit of detection (LOD).

#### Development and fabrication of BSP-loaded PVA MNs

Micro-molding technique was used to fabricate the PVA MNs. Polydimethyl siloxane molds were prepared in-house: silicone elastomer and curing agent were mixed in a 9:1 ratio, poured onto the metal master structures, followed by vacuum drying (15 min) and oven drying (overnight) at 60 °C. Four grades of PVA namely 4–88, 8–88, 18–88, and 26–88 were used to make up the needle-tips. To form the drug-loaded needle tips, BSP was dissolved in the 20% w/w PVA and 10% w/w maltose solution to obtain a drug-polymer solution containing 10% w/w BSP. Further, 100 mg of this solution was weighed into the MN molds, followed by centrifugation at 4000 rpm for 15 min and overnight drying at 60 °C in a convection oven. Hence, the drug loading per MN array was 10 mg of BSP. To form the baseplate, 200 mg of 20% w/w 40–88 PVA solution of a higher molecular weight grade PVA (40–88) was added stepwise onto the pre-dried needle tips, followed by centrifugation at 4000 rpm for 30 min and drying in the convection oven for 2 days, to obtain the final MN arrays. The fabricated MN-TDS were then de-molded and stored in desiccators until further use.

#### FTIR analysis

FTIR analysis (IRAffinity-1 S, Fourier-Transform Infrared Spectrophotometer, Shimadzu Corp, MD, USA) was carried out to check for any molecular interactions between BSP and the PVA grades. All components of the final drug-polymer solutions were analyzed individually (water, BSP, maltose, and 20% w/w solutions of PVA 4–88, 8–88, 18–88, 26–88 in water) and then compared with the polymer solution spectra. Samples were scanned over a range of 4000–400 cm^− 1^ wavenumbers, averaging 100 scans per sample in transmission mode, using the Happ Genzel apodization function. The characteristic peaks for all samples were then evaluated to identify/rule out any potential interactions.

#### SEM imaging

SEM imaging was carried out to evaluate the shape and dimensions of the individual MNs as well as the interspacing within the MN array. The SEM stub was coated with black, non-reflecting double-sided tape. Each MN array was carefully adhered to the tape and loaded into the SEM chamber for analysis. The procured images were then processed to obtain the final measurements of needle height, needle base width, and needle-to-needle distance.

#### Parafilm M® insertion testing

A skin-simulant Parafilm^®^ model was used to test the insertion capability and mechanical uniformity of the fabricated drug-loaded MN arrays. The Parafilm^®^ was folded upto 10 layers, and the MN array was manually applied with uniform pressure for 2 min, followed by careful removal of the array. The treated Parafilm^®^ layers were observed under a Leica DM 750 optical microscope (Leica Microsystems Inc., Buffalo Grove, IL, USA) using the x4 and x10 lens objectives.

#### Histological evaluation

Histological evaluation was performed using hematoxylin and eosin (H&E) staining technique previously described by us [34]. The untreated skin and skin samples treated with drug-loaded MN arrays were embedded in the OCT (optimum cutting temperature) medium and frozen at -80 °C for 20 min. The frozen samples were then adhered to metal stubs using the OCT media and frozen in place. The metal stubs were further loaded onto a Leica CM1860 cryotome. The angle of the blade, stage, and cover slide were optimized to obtain 10 μm thin skin sections over Polysine™ slides (Globe Scientific, Inc., NJ, USA). The tissues were fixed onto the slides using 1% wv formalin solution for 15 min, followed by H&E staining, and preserved in xylene to avoid drying. Slides were examined under a Leica DM 750 optical microscope at x10 lens objective to visualize the intact skin layers versus the micropores created by the MN arrays.

#### Confocal microscopy

Confocal microscopy was carried out to determine the depth of penetration of PVA MNs. The skin samples were treated with the MN array for 2 min, followed by the application of fluorescent dye (Fluoresoft-0.35%^®^) for 2 min. The excess dye was carefully wiped off and the skin samples were then analyzed using a Leica SP8 confocal laser scanning microscope (x10 air lens objective, 496 nm excitation wavelength 496 nm). Z-stacking was carried out with a step size of 10 μm starting from the surface of the skin till the fluorescent dye disappeared from view. The depth of penetration was calculated by considering the step size thickness, multiplied by the number of z-stacks through the samples.

#### In vitro release testing (IVRT)

Cellulose dialysis membrane with a cutoff molecular weight of 14 kDa was used for IVRT testing. BSP, being hydrophilic in nature, was found to be freely soluble in PBS. Hence, PBS was used as the receptor media. The membrane was cut into 1 × 1 inch pieces, pre-soaked in PBS for 20 min, and mounted on vertical Franz diffusion cells (5 mL volume, 0.64 cm^2^ diffusion area) (PermeGear, Inc., Hellertown, PA, USA). The temperature of the receptor chamber was maintained at 35 °C using a circulating water bath system such that the temperature of the membrane was maintained at 32 °C ± 1 °C. Deionized water (300 µL) was added to the donor compartment to function as dissolution media and MN arrays of each PVA grade (*n* = 4) were dipped in this donor solution, with the needles facing upwards to avoid compromising the physical integrity of the membrane structure. The donor chamber was occluded with Parafilm^®^ throughout the study to prevent evaporation of dissolution media from the donor chamber. Samples (300 µL) were withdrawn at predetermined timepoints followed by replenishment with fresh media upto 7 days and analyzed for the concentration of BSP using the validated RP-HPLC method.

#### In vitro permeation testing (IVPT)

##### Skin’s barrier integrity testing

The dermatomed human skin was procured from a tissue bank and was received under dry ice (frozen conditions) and stored at -80 °C till the morning of the IVPT study. The skin was allowed to thaw by placing it (stratum corneum side up) on the benchtop at room temperature for 10 min. Further, the skin was cut into pieces, mounted on vertical Franz diffusion cells, and allowed to equilibrate for 15 min. The barrier integrity of dermatomed human skin was tested using trans-epidermal electrical resistance testing. The detailed procedure has been previously described by us [35]. The electrical resistance across skin samples was measured using a digital multimeter (Agilent Technologies, Santa Clara, CA, U.S.A). Skin samples with a resistance of at least 10 kΩ were used for IVPT testing. For the Dr. Pen Ultima control group (detailed in the next section), the skin’s electrical resistance was measured before and after treatment to ensure successful microporation of the skin samples.

##### IVPT groups

Dermatomed human skin mounted on vertical Franz diffusion cells (5 mL volume, 0.64 cm^2^ diffusion area) (PermeGear, Inc., Hellertown, PA, USA) was used to test the in vitro transdermal permeation of BSP over 7 days. PBS was used as the receptor media. The temperature of the receptor chamber was maintained at 37 °C using a circulating water bath system such that the temperature of the skin was maintained at 32 °C ± 1 °C. Two control groups were used in the study. The first control was untreated/intact skin, and the second control was skin pre-treated with Dr. Pen Ultima (500 μm metal MN device used for 10 s per skin sample). For both the controls, 100 µL of a 10 mg/mL solution of BSP in PBS was dosed. To test the transdermal permeation from the needle-tip-loaded BSP MNs, the arrays were manually inserted into the skin, applying uniform thumb pressure for 2 mins, followed by taping the array in place with 3 M tape, before mounting on the Franz cells. Samples (300 µL) were withdrawn at predetermined timepoints followed by replenishment with fresh receptor media upto 7 days and analyzed using the validated RP-HPLC method. These concentrations were used to obtain the cumulative amount of drug permeated across the skin as well as the permeation profiles for all treatment groups (*n* = 4).

### Statistical analysis

Statistical analysis was carried out using GraphPad Prism (GraphPad Software, San Diego, CA; version 8.0.1) and reported as mean with standard error (in replicates of 4). Ordinary one-way ANOVA was used for the comparison of three or more independent groups, followed by Dunnett’s post hoc test for correcting for multiple comparisons within the group set. A p-value less than 0.05 was considered as the indication of a statistically significant difference between groups.

## Results

### Quantitative analysis

**Fig. 1:**
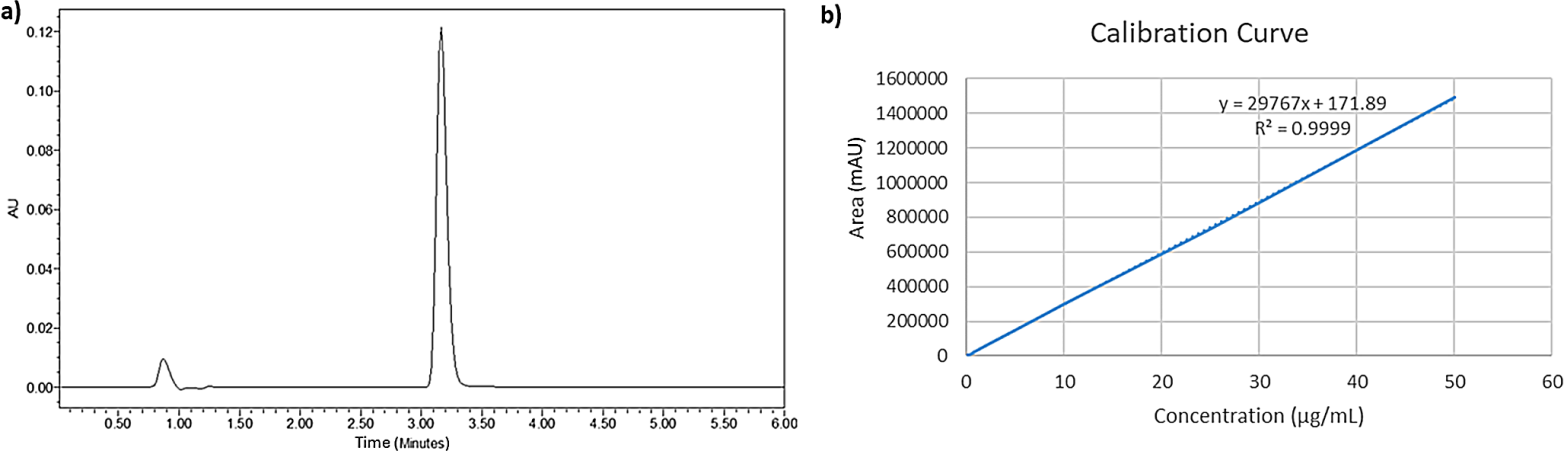
**a**) HPLC chromatogram for a 25 µg/mL concentration of BSP in PBS and **b**) Calibration curve of BSP

The RP-HPLC method for the quantitative analysis of BSP was validated for interday and intraday precision and accuracy. Figure 1.a demonstrates the HPLC chromatogram of 25 µg/mL of BSP. The method was linear (R^2^ of 0.9999), as seen in Fig. 1.b, within a range of 0.1 to 50 µg/mL. The LOD and LOQ were found to be 0.02 µg/mL and 0.06 µg/mL respectively.

### FTIR analysis

**Fig. 2:**
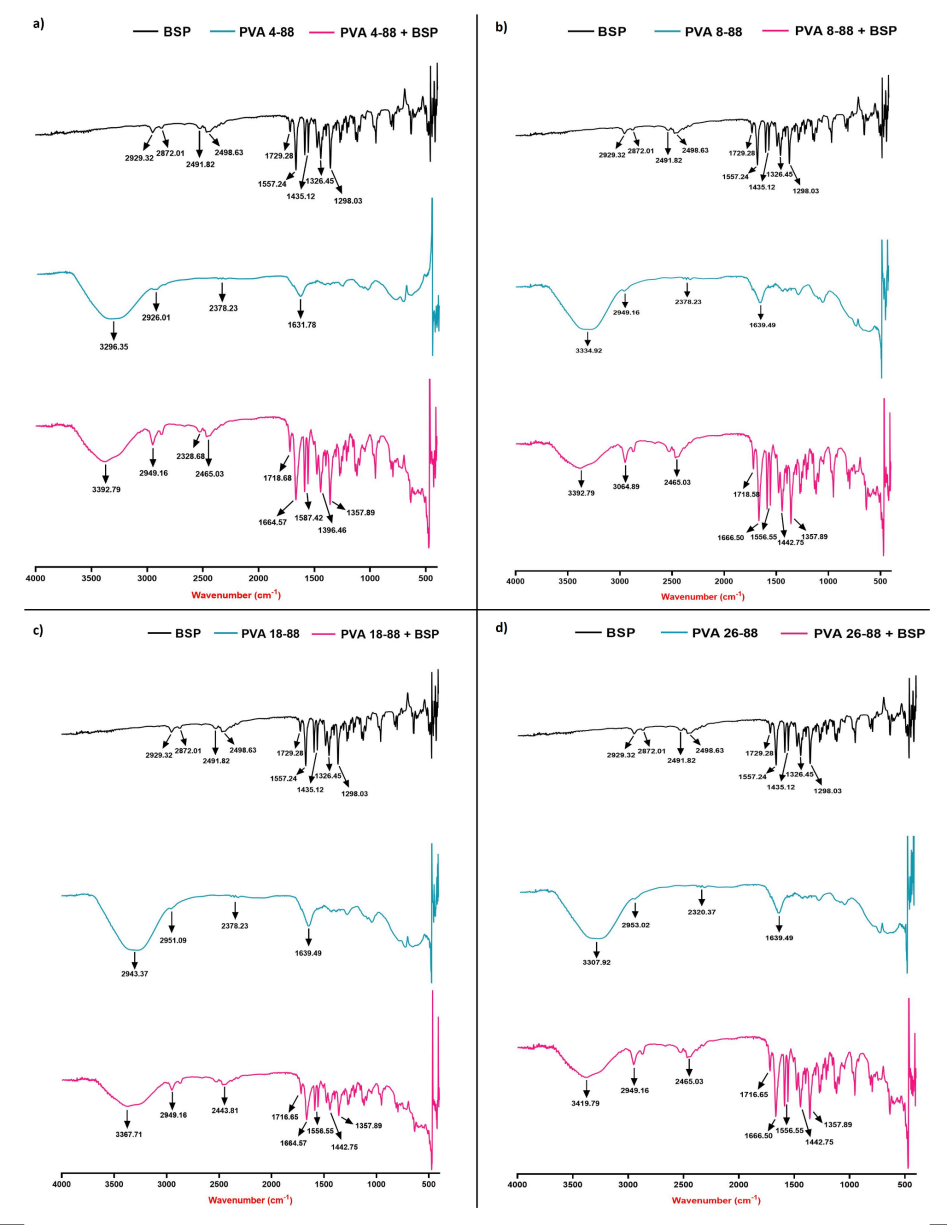
FTIR analysis for the different grades of PVA (**a**) 4–88, (**b**) 8–88, (**c**) 18–88, and (**d**) 26–88

The characteristic functional group peaks for BSP and PVA (20% w/v solution in water) were observed in the FTIR spectra, as seen in Fig. 2. The same has been summarized in Table 1. On analyzing the BSP-PVA solution in water, it was observed that the characteristic peaks of each constituent were retained in the FTIR spectra, thus confirming that the compounds did not undergo any significant change in structure or functional groups. Overlapping of certain peaks was observed due to transmittance at similar wavenumbers. A slightly less broad OH stretching was observed in the BSP-PVA spectra due to overlap around 3000 cm^− 1^ with the C-H of BSP. Similarly, the C = O stretching of BSP appears broader in the BSP-PVA spectra due to overlap with the O-H bending vibration of PVA adsorbed water molecules (PVA in solution).

**Table 1:** Characteristic FTIR peaks for various functional groups in BSP and PVA

Compound	Wavenumber range (cm^− 1^)	Functional groups
BSP	1650–1700	C = O stretching
	2850–3000	C-H aromatic and aliphatic
	1500–1600	C = C stretching
	1200–1350	C-N stretching
PVA	3200–3600	O-H broad stretching
	2800–3000	C-H aliphatic stretching
	1600–1650	O-H bending vibration associated with adsorbed water molecules

### SEM

**Fig. 3:**
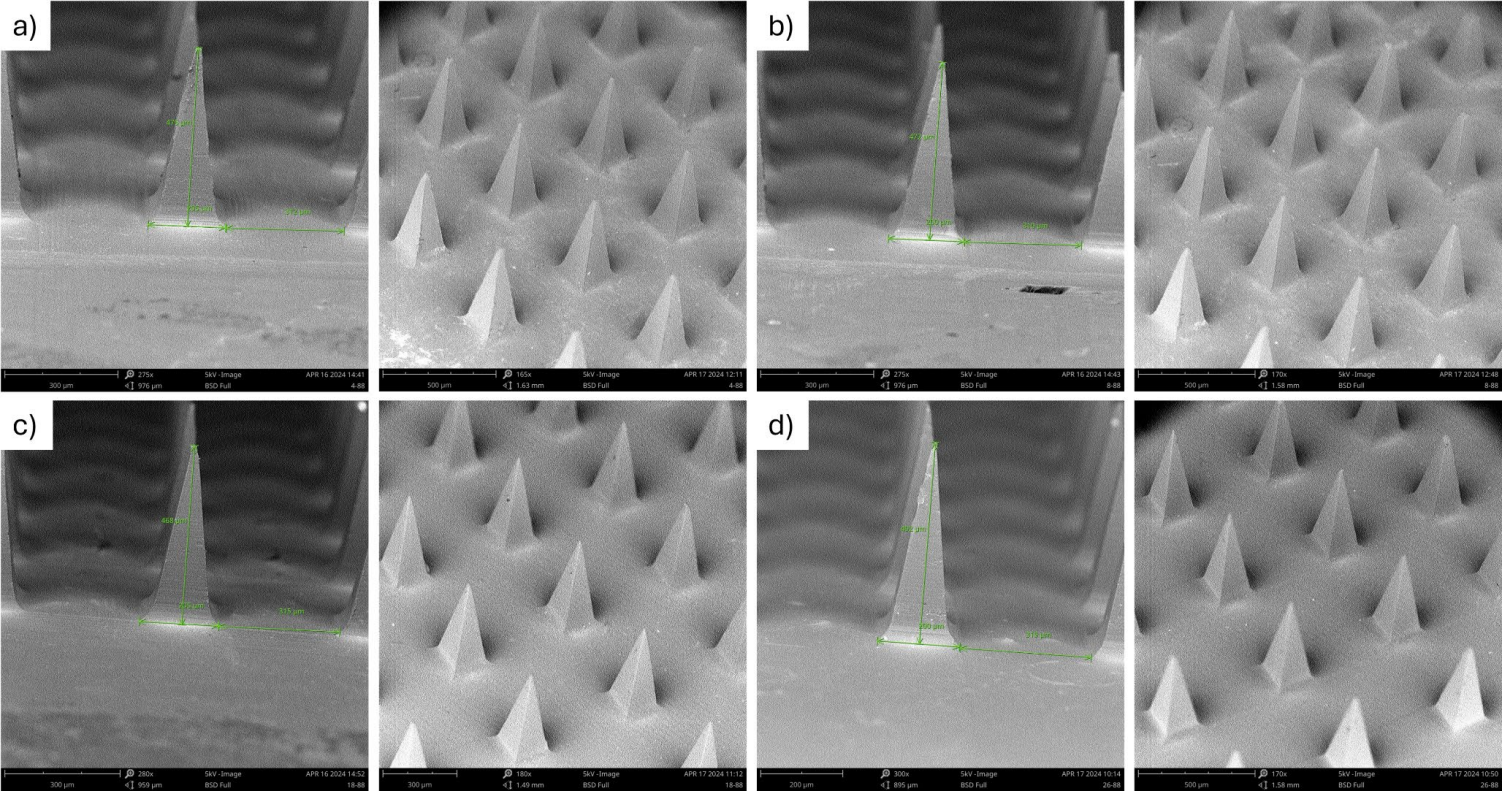
SEM images (**a**) 4–88, (**b**) 8–88, (**c**) 18–88, and (**d**) 26–88

SEM imaging was carried out to evaluate the shape and dimension of the individual MNs as well as the array geometry. The square pyramidal-MN arrays were found to be structurally uniform (Fig. 3). The height and base width of the MNs and the interspacing distance between two adjacent MNs (distance between the centers of each base) were calculated in triplicate, the results were as shown in Table 2.

**Table 2:** Needle height, needle base width, and interspacing distances for BSP-PVA MN-TDS

Needle tip PVA polymer	Needle height (µm)	Needle base width (µm)	Interspacing distance (µm)
4–88	483.00 ± 4.16	208.67 ± 2.03	311.67 ± 0.88
8–88	483.00 ± 5.68	206.00 ± 3.21	311.67 ± 1.20
18–88	481.33 ± 6.76	205.00 ± 1.73	311.00 ± 2.31
26–88	485.33 ± 4.41	208.67 ± 4.37	315.67 ± 1.76

### Parafilm® insertion testing

**Fig. 4:**
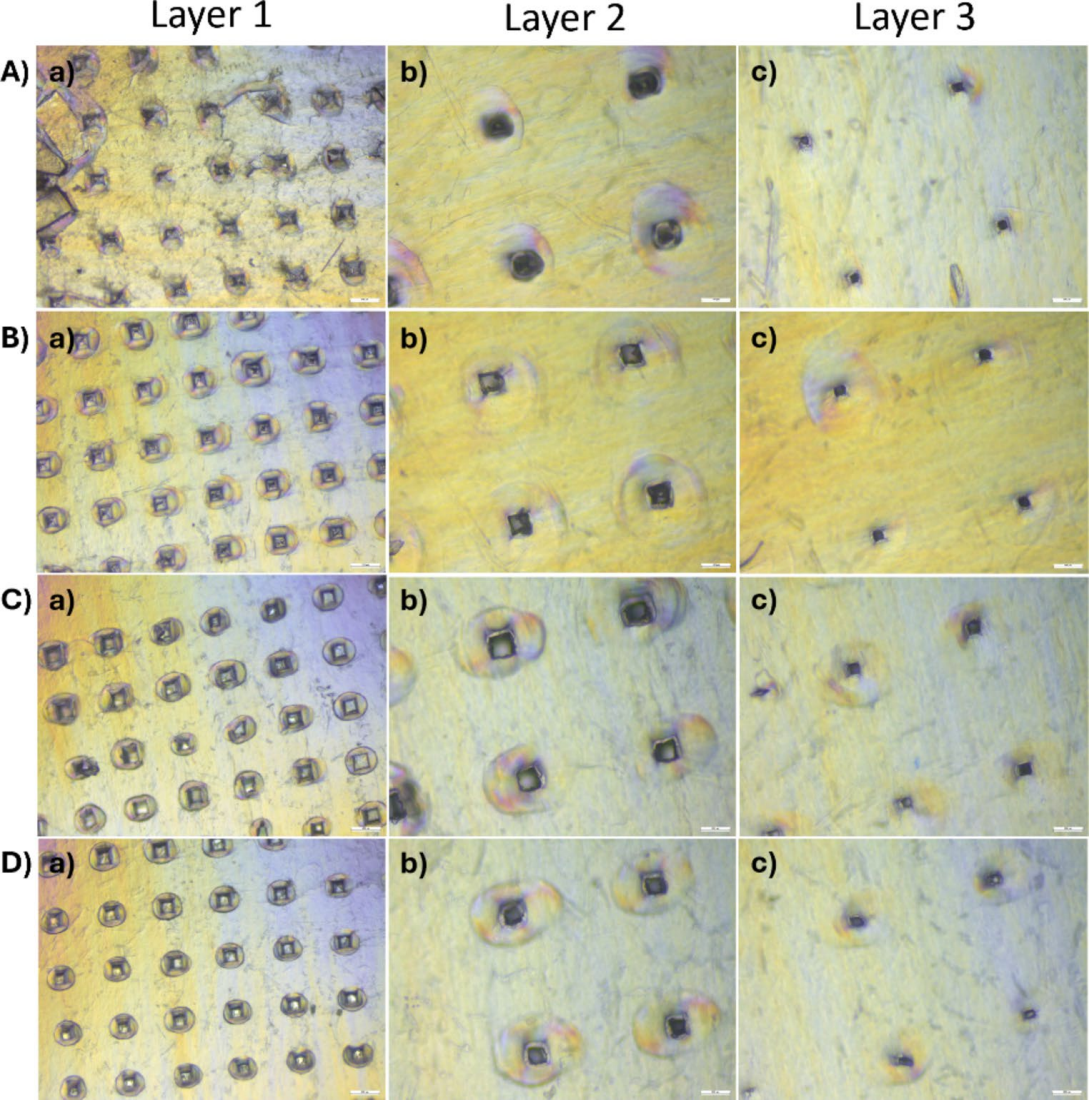
Parafilm^®^ insertion testing as a skin-simulant model for BSP-loaded MNs **A**) 4–88, **B**) 8–88, **C**) 18–88, and **D**) 26–88. Scale bar denotes 100 μm

Parafilm^®^ was used as a skin simulant model due to its elasticity and flexibility. Upon treatment with BSP-loaded MN-TDS and observation under the microscope, it was seen that the MN arrays penetrated 3 layers of parafilm (Fig. 4). The microchannels were observed to be uniform within the array as well as across all grades of PVA. The three layers subsequently had smaller microchannels as the MNs passed through them.

### Histological evaluation

**Fig. 5:**
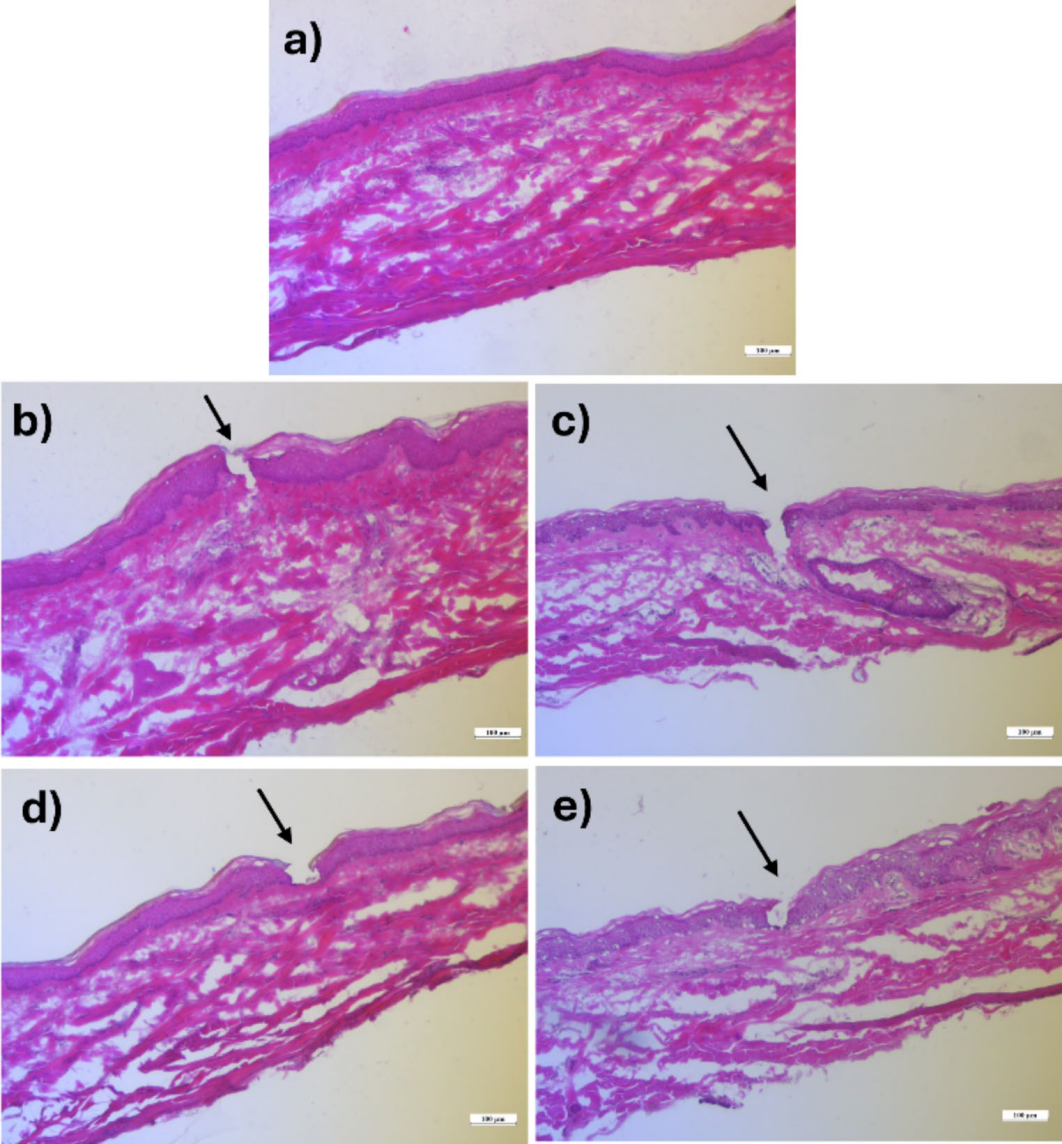
Histological evaluation of **a**) untreated skin and MN-treated skin **b**) 4–88, **c**) 8–88, **d**) 18–88, and **e**) 26–88. Scale bar denotes 100 μm

Histological evaluation was carried out to observe the formation of micropores after treatment with MNs. Figure 5.a shows an untreated skin sample with intact stratum corneum, epidermis, and dermis layers. For the skin samples treated with BSP-loaded MN arrays (also shown in Fig. 5), micropores were observed puncturing the stratum corneum and extending into the epidermal as well as upper dermal layers of the skin. No visible differences in penetration ability were observed between the different types of PVAs in the fabricated MN-TDS.

### Confocal microscopy

**Fig. 6:**
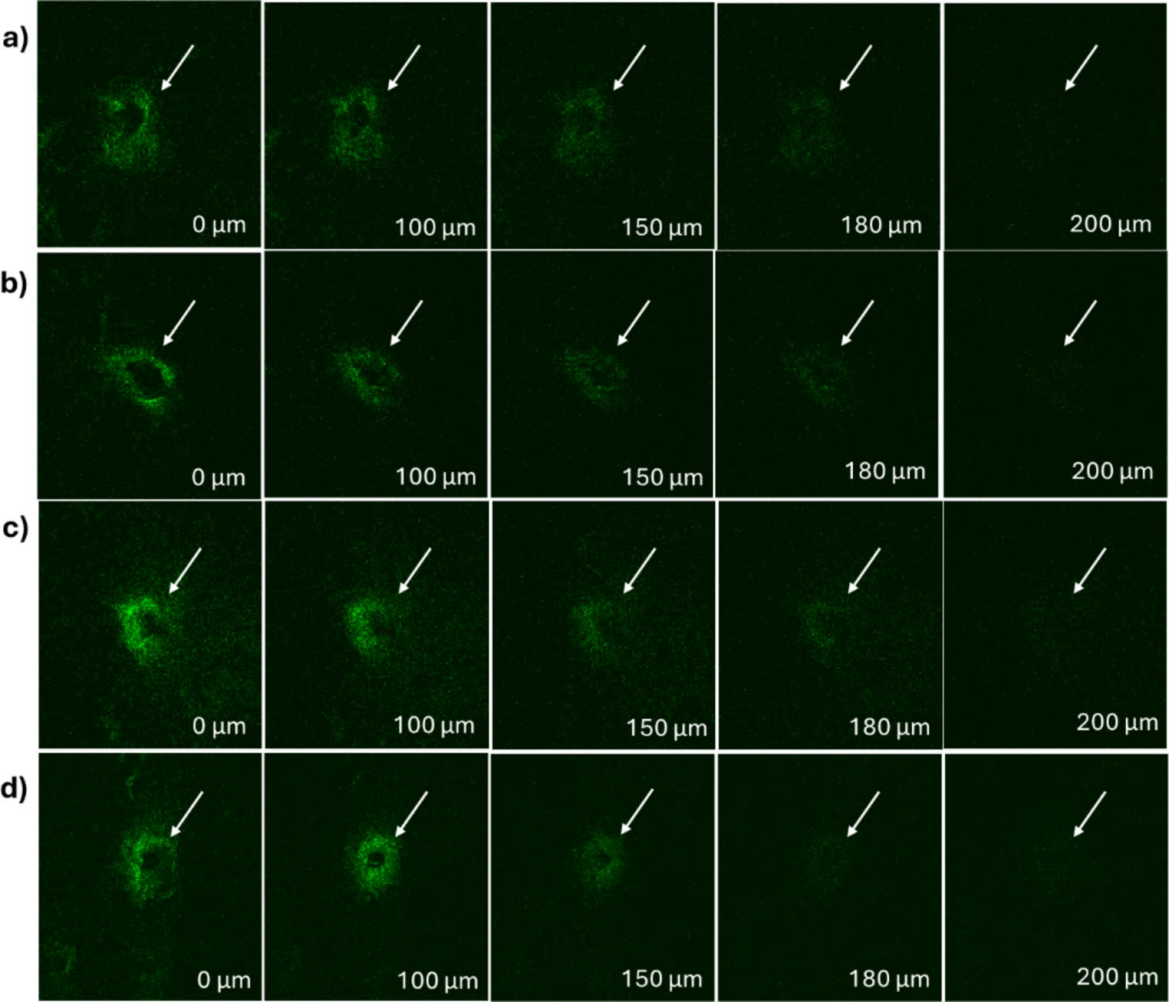
Confocal microscopy of human skin treated with BSP needle-tip loaded MNs fabricated using different PVA grades **a**) 4–88, **b**) 8–88, **c**) 18–88, and **d**) 26–88

Confocal microscopy was carried out to evaluate the depth of penetration of the drug-loaded PVA MN-TDS into dermatomed human skin, as seen in Fig. 6. All the fabricated PVA MNs had approximately the same penetration depth of 200 μm. Hence, no differences in the penetration depth was observed within the PVA grades.

### IVRT studies

**Fig. 7:**
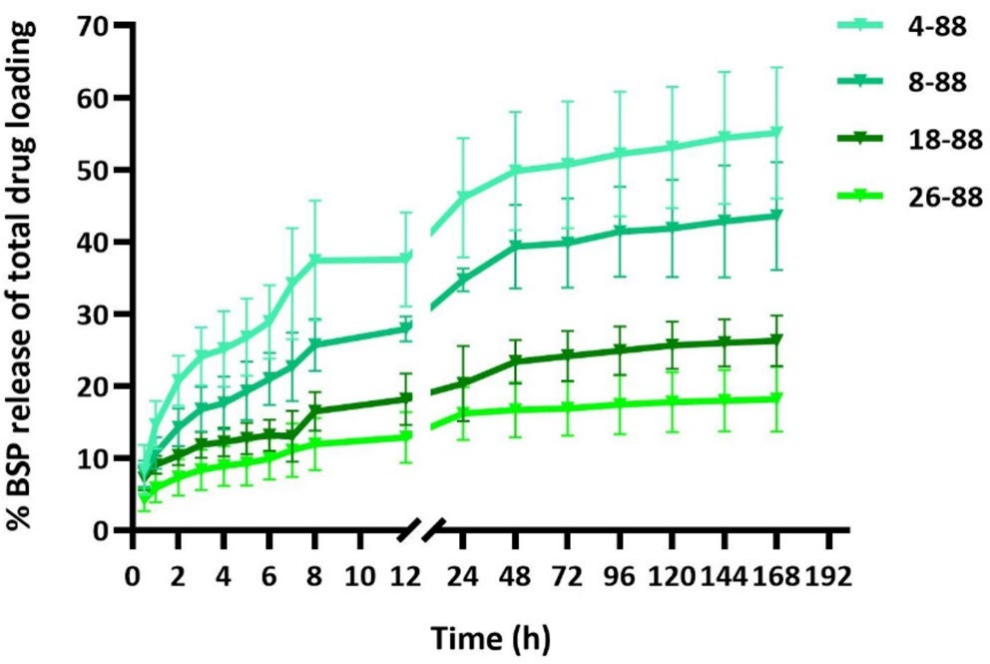
IVRT results: Release profiles for BSP needle-tip loaded PVA MNs over a 7-day duration across cellulose dialysis membrane

IVRT studies were conducted to observe the release pattern of BSP from the PVA MNs over a sustained period of 7 days. It was observed that the lower the molecular weight of the PVA, higher the cumulative release of BSP. The PVA 4–88 MNs released 55.07% of the total drug loaded (5507.37 ± 456.88 µg/cm^2^), followed by 43.59% from PVA 8–88 (4359.56 ± 374.93 µg/cm^2^), 26.27% from PVA 18–88 (2627.34 ± 176.66 µg/cm^2^), and only 18.17% from PVA 26–88 (1816.81 ± 224.82 µg/cm^2^). Moreover, it was observed that nearly 80% of the total drug released within 7 days was released in the first 24 h of the IVRT. As seen in Fig. 7, this rapid release was then followed by a steady release for the next 6 days.

### IVPT studies

**Fig. 8:**
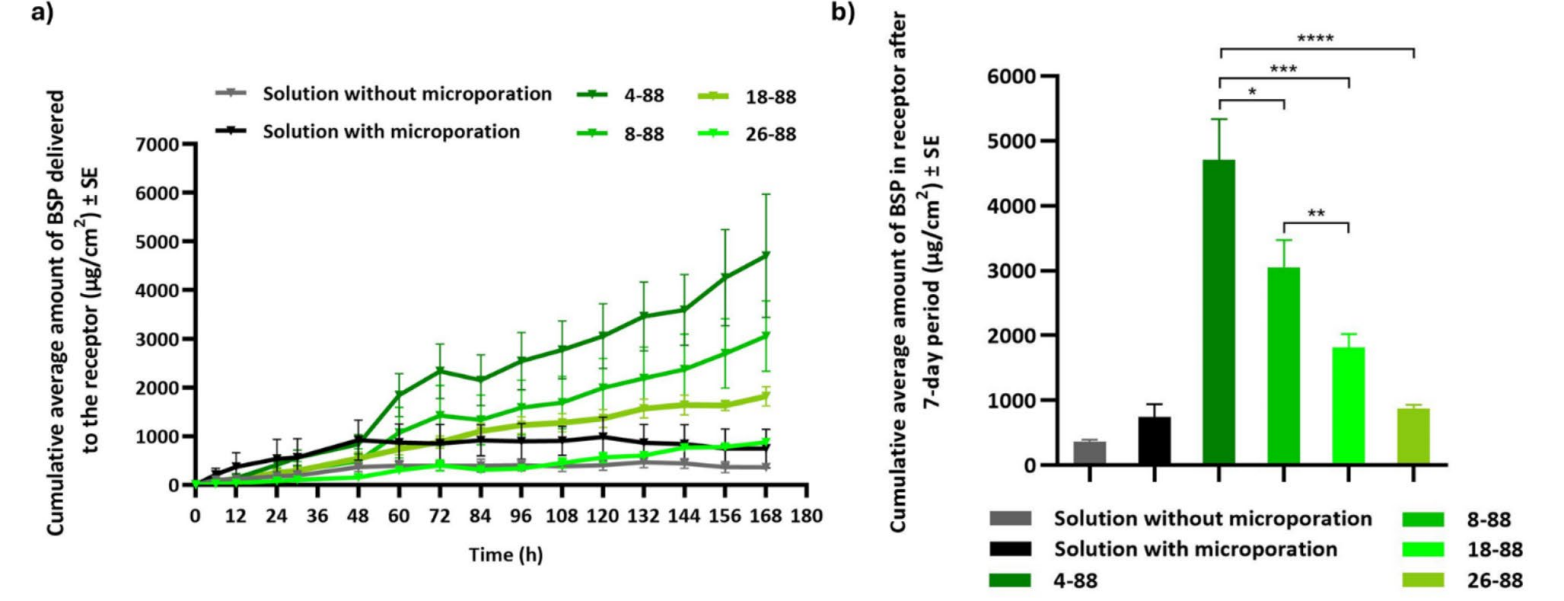
IVPT Results: Comparison of transdermal delivery of BSO from different PVA MNs **a**) Permeation profile and **b**) Cumulative transdermal delivery at the end of 7-day study duration. Statistical analysis using one-way ANOVA *p < 0.05, **p < 0.01, ***p < 0.001, ****p < 0.0001

IVPT studies were conducted across dermatomed human skin to evaluate the transdermal delivery of BSP from various PVA MNs (*n* = 4). Control groups were also evaluated (*n* = 4), where a solution of BSP was dosed with and without pre-treatment with a metal microneedle device (Dr. Pen Ultima). As seen in Fig. 8, the control groups showed a drop in permeation after 48 h. Moreover, the cumulative delivery over 7 days for both the control groups was lower than the PVA MN groups (360.54 ± 29.86 µg/cm^2^ and 744.55 ± 197.13 µg/cm^2^ without and with microporation, respectively). On the other hand, the fabricated BSP needle-tip loaded PVA MNs showed sustained delivery profiles over the 7 days. Depending on the grade of PVA used, the cumulative transdermal delivery varied significantly, where the rate and extent of permeation were inversely proportional to the molecular weight of the PVA used in the needle tips. Hence, PVA 4–88 had significantly higher total delivery amounting to 47.05% of the drug loading (4705.42 ± 634.57 µg/cm^2^), followed by 30.52% delivery from PVA 8–88 (3051.93 ± 418.15 µg/cm^2^), 18.19% from PVA 18–88 (1818.94 ± 201.39 µg/cm^2^), and only 8.75% from PVA 26–88 (874.47 ± 57.78 µg/cm^2^).

## Discussion

The global valuation for transdermal MNs is projected to cross $10.9 billion by 2033, as per recent market reports [36]. MN-TDS is, therefore, a well-funded and actively researched delivery system with multiple products in clinical trials [37]. MNs hold significant advantages over conventional oral and parenteral treatment approaches while addressing certain drawbacks. They offer high drug bioavailability, ease of self-administration, and a painless, non-invasive route of systemic drug administration. Specifically, long-acting MN-TDS offers reduced dosing frequency and fluctuations in systemic drug levels, as well as improved patient compliance and adherence to treatment regimes over long durations. The present study focused on developing polymeric MNs as a sustained TDS of BSP for the management of anxiety disorders. Micro-molding is the most commonly used fabrication technique, especially for dissolving MNs. This technique is highly reproducible and cost-effective, allowing versatility in the construction material. It also provides high precision, ease of scalability, and reasonable design flexibility without requiring costly equipment [21]. In the present study, micro-molding technique was utilized for the fabrication of MNs using various PVA grades. PVA MNs have been previously reported for long-acting transdermal delivery of various molecules. Tekko et al. loaded cabotegravir hydrogel in PVA-PVP MNs whereas Mc Crudden et al. designed a nanosuspension formulation within PVA MNs to achieve sustained delivery of rilpivirine [38, 39].

In the present study, four different grades of PVA namely 4–88, 8–88, 18–88, and 26–88 were utilized in the needle-tips of the MN structures. To adequately sustain the drug release from the MNs, a higher % of PVA was prioritized while optimizing the formulation process. BSP-containing needle-tip polymer solutions were dispensed into the PDMS molds and allowed to dry overnight before the step-wise addition and drying of the baseplate polymer solution. Vacuum drying (at approximately 20 mBar) and centrifugation were explored as techniques to facilitate the passage of the viscous needle-tip solutions into the micro-molds. It was observed that vacuum-drying created air pockets, leading to incomplete formation of the needle-tips. In contrast, centrifugation was able to eliminate the entrapment of air bubbles in the MN structure. Consequently, vacuum drying was also explored for preparation of the baseplate, but resulted in a porous baseplate lacking mechanical strength. Hence, centrifugation followed by oven drying was carried out for the fabrication process. Since we aimed to compare the different grades, the concentration of PVA was kept constant across the different grades during the trials/optimization of the formulation. Upon testing different PVA concentrations, it was found that a 20% w/w solution across all PVA grades resulted in well-formed MNs. Concentrations higher than 20% w/w formed incomplete arrays due to the viscosity of the polymer solutions. Although the 20% w/w PVA MNs were well-formed, they were found to be brittle during preliminary characterization testing. Hence, 10% w/w of maltose was added to the polymer solutions to improve the flexibility of the MN structure. Maltose has been previously reported to support the mechanical strength of PVA MNs [40, 41].

We aimed to load BSP only in the needle-tips to avoid drug wastage. A major hurdle for needle-tip loaded MNs is the potential backflow or migration of the drug from the needle-tips to the baseplate during the formulation process. This results in an inadequate amount of drug available in the needle-tips for transdermal delivery. To address this drawback, we dispensed the BSP-containing aqueous needle-tip polymer solution in the MN mold, and let it dry completely, before adding the baseplate polymer solution. Secondly, we used a higher M_w_ PVA (40–88) for the baseplate than all the needle-tip PVA grades, to restrict the backflow of BSP. Moreover, we also tested various organic solvents, and found that N-methyl pyrrolidone (NMP) solubilized PVA, but was a non-solvent for BSP. Hence, we tried to use NMP to prepare the baseplate polymer solution, to further prevent BSP backflow. However, the formed baseplate detached from needle-tips during fabrication, along with visible clumps/precipitates, signifying incompatibility. Consequently, water was used as the solvent for the baseplate polymer solution. Among the PVA grades explored, all of them had the same % of hydrolyzed acetate ester groups = 88%. Partially hydrolyzed PVAs (87–89%) have higher solubility in water, more flexibility, and better adhesion to hydrophobic surfaces, as compared to the highly hydrolyzed PVA (91–99%) [27, 42]. The explored grades differed in their M_W_ in the ascending order of 4–88, 8–88, 18–88, and 26–88. The solubility of PVA in water decreases with increasing M_W_ [30]. All the explored grades of PVA were completely soluble in water at 20% w/w concentration and were further able to solubilize 10% w/w of BSP. FTIR studies confirmed that the key functional groups of PVA and BSP were intact; no significant molecular interactions existed between them. Hence, PVA acted as an inert polymer for the fabrication of BSP MNs.

The fabricated MNs were subjected to various characterization studies to identify any differences among the PVA grades. It was found that the PVA grades did not exhibit any significant differences in their appearance, penetration ability (Parafilm^®^ as well as dermatomed human skin), or overall mechanical integrity. According to the SEM imaging, all four types of MNs were structurally sound. Parafilm^®^ testing demonstrated penetration ability and mechanical strength of the MNs. Histological evaluation confirmed the successful disruption of the skin’s layers, whereas confocal microscopy identified the depth of penetration of the MNs. Hence, all four types of MNs were further explored using in vitro performance tests.

IVRT studies were carried out using a dialysis membrane, to observe the drug release patterns from the PVA MNs. The most commonly reported method of testing drug release from MNs includes suspending the MNs within a membrane pouch inside a continuously stirred beaker apparatus [43]. However, this method involves rapid movement of the MN-TDS and ambiguity regarding the homogeneity of drug concentration throughout the beaker. Moreover, an infinite volume of receptor media is available for dissolution, which does not correlate well with in vivo settings where the amount of interstitial fluid within the skin layers is finite. In the present study, the membrane was mounted onto Franz diffusion cells, and the MNs were suspended in a finite amount of release media inside the donor chamber. This allowed the released drug to freely permeate across the membrane and into the receptor compartment of the Franz cell, from where the drug amounts were analyzed. As compared to previously reported methods, the current method prevents movement of the TDS itself and also ensures uniform mixing of the samples receptor media due to the Franz cell setup. The IVRT study was conducted over a 7-day period, where a burst release was observed within the first 12 h, followed by sustained release over 7 days. This can be correlated with the finite availability of the release media, which may have led to a shift in the concentration gradient and possible saturation of the media in the donor chamber after 12 h. It was also seen that the rate and extent of drug release was inversely proportional to the M_W_ of the PVA. This can be correlated to the decreasing solubility of higher M_W_ PVAs, thus impacting the dissolution of the MNs and hence the drug release pattern. At the end of the study, it was observed that the MNs had achieved a rubbery, rigid gel-like consistency within the donor chamber. Moreover, only 18–55% of the total loaded BSP was released within 7 days, signifying that the drug release could be potentially sustained past the 7-day study duration.

IVPT studies were carried out to understand the permeation of BSP across human skin. The first control group, in which a BSP solution was administered onto intact human skin, showed that the permeation profile could not be sustained past the 48-hour mark. In the second control group, in which skin was microporated before administration of a BSP solution, the permeation values were higher than the first control group, signifying that microporation can successfully enhance transdermal delivery of BSP. But this permeation profile also dropped past the 48-hour mark. Moreover, it is know that pore closure occurs within 15 h of microporation without occlusion and within 72 h with occlusion [44]. Polymeric MNs can be employed to keep the micropores open and facilitate sustained transdermal delivery. The IVPT results showed that the permeation flux was maintained across the 7-day study duration for all the PVA grades. Here, too, the rate and extent of permeation were inversely proportional to the PVA’s M_W_. We calculated the target transdermal delivery to further understand the feasibility of using the fabricated MNs in a clinical setting. Depending on disease severity, BSP is prescribed as multiple daily oral doses upto 60 mg per day. Considering 4% oral bioavailability, 2.4 mg of the maximum oral dose can reach the systemic circulation and exert a therapeutic effect in the human body. Further, considering a 50 cm^2^ application area of a transdermal system (the maximum area of a transdermal patch on the market), the required transdermal delivery for therapeutic effect will be 48 µg/cm^2^ per day, and a cumulative of 336 µg/cm^2^ over a week’s period. Comparing these values to the cumulative transdermal delivery from the PVA MNs, we found that all the PVA grades were able to cross the daily and weekly target delivery of BSP. Moreover, since only 8% t 47% o the drug loading was delivered over 7-days, the dosing period can be extended past this duration to reduce the dosing frequency further. Moreover, the data generated regarding the trends of drug release and permeation from various PVA grades can be potentially extrapolated to develop MN-TDS for hydrophilic molecules.

## Conclusion

The present study described the fabrication of needle-tip-loaded PVA MNs of BSP using micro-molding technique. Four types of PVA were utilized to form sharp, well-formed, and mechanically resilient MN arrays. Upon evaluation and comparison of the fabricated MNs using various characterization tests, it was found that the PVA grades did not result in differences in MN geometry, mechanical strength, or insertion capability of the MNs. In vitro performance testing established that the PVA grades significantly affected the rate and extent of drug release and permeation across human skin. Further, all types of PVA grades could cross the daily and weekly transdermal delivery target for the systemic delivery of BSP. Overall, the study demonstrated the feasibility of sustained transdermal delivery of BSP over 7 days for the management of anxiety disorders using the fabricated needle-tip-loaded PVA MNs.

## Abbreviations

PVA: Poly-vinyl alcohol
MNs: Microneedles
ADHD: Attention-deficit/hyperactive disorder
BSP: Buspirone hydrochloride
MN-TDS: Microneedle transdermal delivery systems
SSRI: Selective serotonin reuptake inhibitors
SEM: Scanning electron microscopy
LOD: Limit of detection
LOQ: Limit of quantitation
IVRT: In vitro release testing
IVPT: In vitro permeation testing
PBS: Phosphate-buffered saline
